# Artificial Intelligence in the Management of Polypharmacy Among Older Adults: A Scoping Review

**DOI:** 10.7759/cureus.90867

**Published:** 2025-08-24

**Authors:** Kyle Bringhurst, Talaya Jones, Gerard Runko, Matthew Jabbari, Nicoletta Zipparro, Gia Nghi Vo, Aman Ullah, Tri Minh Vo, Maxwell Corrigan, Vince Birrey, Robin J Jacobs

**Affiliations:** 1 Dr. Kiran C. Patel College of Osteopathic Medicine, Nova Southeastern University, Fort Lauderdale, USA

**Keywords:** ai, drug-drug interaction, geriatric, health technology, large language model (llm), machine learning (ml), multiple medication, older adults, patient management, pharmaceutical management

## Abstract

The advent of artificial intelligence (AI) presents an opportunity to enhance the management of multiple medications for patients. Despite the growing body of research on the clinical impact and accuracy of AI in healthcare, current literature is limited in addressing AI’s role in medication management for older adults. The goal of this scoping review was to assess the extent and applicability of AI applications in managing multiple pharmacological therapies among adults aged 50 and older. Relevant articles were searched in EMBASE, Ovid MEDLINE, and Web of Science databases. Search phrases included “polypharmacy/polytherapy”, “artificial intelligence in healthcare”, “machine learning/language learning”, and “elderly populations”. The search initially identified 58 citations. After a systemized and rigorous screening process, 12 articles were further screened for eligibility and critically appraised for bias and appropriateness. Of those, a total of five articles were retained for the final analysis. They focused on the application of AI and web-based applications to reduce inappropriate medication use and drug interactions, how machine learning and audio-based activity recognition systems could improve medication adherence among older adults, and recognizable patterns between multimorbidity and polypharmacy in elderly populations with chronic illnesses. The main findings of this review suggested that AI tools have demonstrated efficiency and accuracy in eliminating drug-drug interactions, assisting in the detection of potentially inappropriate medications (PIMs), and identifying patterns of multimorbidity due to polypharmacy in older adults. Moreover, AI tools were considered easy to use and helped enhance medication adherence. Results suggest that AI has potential in managing polypharmacy, particularly in enhancing medication safety, improving adherence, and predicting risk factors for medication-related errors in older adults. Future investigations are needed that focus on AI’s long-term effect on patient outcomes, its role in personalized pharmacotherapy, and ongoing challenges related to algorithmic transparency, bias mitigation, and regulatory oversight. As AI continues to evolve, its integration into healthcare would benefit from methodical evaluation, stringent oversight, and interdisciplinary collaboration to ensure its safe and effective deployment in patient care.

## Introduction and background

Prescribing medications has increased markedly in recent years, contributing to global health challenges including higher medical care costs, a rise in opioid-related mortality, an increased prevalence of addiction disorders, and a lack of comprehensive strategies to mitigate these adverse outcomes [1]. Older adults remain a particularly vulnerable population, as they are more likely to require multiple management medications and complex regimens, leading to elevated risks of inappropriate use and subsequent complications [2].

The advent of artificial intelligence (AI) presents an opportunity to enhance the management of multiple medications, particularly by improving the understanding of drug interactions, predicting adverse outcomes, and assisting patients with medication adherence. This technological advancement is especially pertinent given the physiological changes that accompany aging, which can

significantly affect how the body absorbs, distributes, metabolizes, and eliminates medications. For instance, the decline in liver and kidney function commonly seen in older adults can slow drug clearance, increasing the risk of toxicity. Additionally, age-related changes in body composition, such as increased fat and decreased muscle mass, further influence drug distribution and efficacy. These changes, combined with the prevalence of chronic conditions, render older adults more susceptible to morbidity and mortality due to drug-related harms [3].

Polypharmacy, or the use of multiple medications, is prevalent among older adults due to the high incidence of chronic conditions within this population. This practice significantly raises the risk of drug-drug interactions (DDIs) and adverse effects, particularly when over-the-counter medications are taken in conjunction with prescription drugs [3]. Unfortunately, current guidelines for medication management often focus on single diseases while neglecting the complexities introduced by comorbidities and the use of multiple medications [3].

Age-related changes in receptor sensitivity and drug metabolism can heighten older adults’ responsiveness to medications, often leading to more pronounced side effects or adverse reactions - even at lower doses - compared to younger individuals. This heightened sensitivity amplifies the risks associated with DDIs, particularly in older patients with multiple comorbidities, who are often exposed to a large variety of medications [2].

Cognitive decline and other functional impairments that some older adults experience can also impact their ability to manage medications safely, increasing the risk of non-adherence or medication errors. Physical pain is a leading reason for adults seeking medical care, with significant implications for individual well-being and healthcare systems [4]. Pain medications, especially opioids and non-steroidal anti-inflammatory drugs (NSAIDs), are associated with increased risks of falls and fractures in this population due to their effects on balance, coordination, and bone health [5]. Moreover, the decline in liver and kidney function with age further complicates the safe use of these medications, as impaired organ function can lead to the accumulation of drugs and a higher risk of toxicity.

These considerations underscore the importance of a careful and individualized approach to prescribing and managing medications in older adults. This often requires dose adjustments, close monitoring, and a concerted effort to minimize risks while effectively managing symptoms. The integration of DDI prevention into a comprehensive medication action plan that also addresses drug-disease interactions, high-risk therapies, and dosage adjustments may be necessary to account for age-related changes in organ function among older patients [2]. Such a plan is critical for improving the safety and efficacy of pharmaceutical management in the older adult patient population.

Artificial intelligence (AI) and pharmacology

AI presents a promising tool for analyzing complex problems and generating solutions, content, and questions. In the context of pharmacology, AI’s utility in medical education is demonstrated by employing ChatGPT, which is primarily used for natural language understanding and generation, to create multiple-choice exam questions (often labor-intensive when done by hand) [6]. ChatGPT-generated questions exhibited psychometric properties that met acceptable standards [6]. While AI shows promise in pharmacological medical education, AI is continually developing its accuracy and usefulness for such applications.

The integration of AI into medical practice presents several challenges, including the potential for depersonalization of care, susceptibility to errors, the need for human oversight, and the demands for infrastructure such as data storage and reliable server connections. ChatGPT has demonstrated utility in identifying general DDIs and providing basic management recommendations. It may also assist pharmacists with workflow tasks such as clinical documentation and summarization.

For example, a physician might engage ChatGPT by asking, “What are the potential drug-drug interactions between apixaban and fluconazole?” ChatGPT would return a response describing known pharmacokinetic interactions, associated risks (e.g., increased bleeding), and general monitoring recommendations. However, the need for caution and expert oversight when using AI to ensure accuracy and relevance is important [6]. While it shows potential in supporting polypharmacy management across various clinical contexts, its capacity to recommend specific prescription medications or alternative therapies remains limited [7]. Nevertheless, AI’s capabilities are rapidly advancing, and the belief that its intelligence will match humankind’s may soon no longer be considered far-fetched.

Machine learning (ML) and polypharmacy

The significant advancements in machine learning (ML), particularly in large language models (LLMs), have spurred broader discussions regarding their application in medical care. ML is a type of AI wherein computer programming allows the system to learn from previous input through applied algorithms [8]. For example, ML can be used by clinicians to predict patient medication and use behaviors to help optimize care [9]. LLMs are another type of AI (e.g., ChatGPT) that utilize designed algorithms specific to digesting language, often from text, to generate responses and answers from curated banks of information, external or internal to the LLM interaction itself [10].

Pharmaceutical management and older adults

Despite the growing body of research on the clinical impact and accuracy of AI in healthcare, current literature is limited in addressing AI’s role in medication management for older adults. For instance, Rao and colleagues investigated AI's responses to geriatric patients’ medication alterations, while examining dosing accuracy without focusing on the dynamic needs of the geriatric population [9, 11]. Bories et al. highlighted the higher risk of DDIs among older patients in hospital settings compared to primary care and nursing homes [2].

This review addressed the increasing complexity of medication management among older adults due to physiological changes, polypharmacy, and the increasing prevalence of comorbidities. Currently, pharmaceutical management strategies are often insufficient due to the elevated risk of adverse DDIs. While AI may offer innovative solutions to multi-pharmacological management, safety, accuracy, and efficacy in this domain remain unstudied. The goal of this scoping review was to comprehensively summarize the extent of the current literature on AI’s role in multi-pharmacological management, including any unique challenges faced in the treatment of older adults. This scoping review sought to answer the question: How accurate is AI in managing multiple pharmacological therapies for older adults? A preliminary search of MEDLINE, the Cochrane Database of Systematic Reviews, and Joanna Briggs Institute (JBI) Evidence Synthesis was conducted to assess the current systematic reviews or scoping reviews on the topic. This preliminary search yielded no similar reviews related to this project’s scope.

## Review

Methods

This scoping review was conducted using the JBI methodology for scoping reviews to investigate and summarize the published literature on the applicability of AI applications in managing multiple pharmacological therapies among adults aged 50 and older [12]. Peer-reviewed articles published between 2020 and 2024 were searched using academic library databases. Descriptive observational studies (including retrospective observational studies and descriptive cross-sectional studies) were included.

The search was guided by the review question “What is known about the accuracy and safety of AI applications in managing polypharmacy among older adults?”

Eligibility Criteria

The inclusion criteria specified that the articles garnered from the database searches must involve older adults and the management of multiple pharmacologically based treatment plans for this group. For this scoping review, an older adult is defined as aged 50 and older. To ensure consistency and prevent translation errors, only studies written in English were used. To adequately investigate the applicability of AI in polypharmacy interventions for older adults, articles detailing the management of such interventions were included. The exclusion criteria removed studies that examined AI applications in polypharmacy for populations outside the older adult range, defined as 50 and older. Additionally, research focusing exclusively on non-pharmacological symptom management methods was excluded to ensure consistency.

Search Strategy

The Population, Concept, and Context (PCC) framework guided the search strategy. The population was defined as adults 50 and older, the concept was polypharmacy, and the context was the applicability of AI. Data collection using search databases EMBASE, Ovid MEDLINE, and Web of Science (Science Core Collection) was conducted on September 15th, 2024. Search terms included “polypharmacy/polytherapy,” “artificial intelligence in healthcare,” “machine learning/language learning”, and “elderly populations”. Keywords were based on the PCC criteria to yield the most concise and relevant data. The authors used a variety of combinations of keywords to yield greater results and appropriate synonyms. Boolean operators AND for the concurrent occurrence of subjects and OR for the occurrence of their corresponding synonyms were used. Table 1 in the appendix depicts the systemized search strategy used with each database.

Screening and Article Selection

The initial search identified 59 citations. After removing 1 duplicate article, 58 articles remained for further screening. Two research team members screened every article’s title and abstract, reaching a consensus on 12 relevant articles for further full-text screening. Two additional team members reviewed each full article (n = 12) using a screening form to verify inclusion criteria for each article. In the event of a disagreement between team members regarding the eligibility of an article, a third independent reviewer joined the discussion until an agreement was reached. This resulted in the further exclusion of 7 articles for the following reasons: did not use the correct study design, were not available as full-text, or did not meet all of the inclusion criteria. Of the remaining four included articles, a reference in one of the articles was further investigated and found to be eligible and was added, resulting in a total of five articles for the final review.

Data Charting and Extraction Process

Rayyan, a web-based software tool, was utilized to support the screening and selection stages of the scoping review. Microsoft Excel (Microsoft Corporation, Redmond, WA) was employed for data extraction and charting. Data management throughout the review followed an iterative approach, with team members independently charting data, engaging in discussions to interpret findings, and continually refining the data-charting form. Extracted information centered on each article’s country, aims, study population, method, limitations, results, and conclusions from the final set of included studies.

Critical Appraisal of Individual Sources of Evidence

The JBI Critical Appraisal Tools were used to evaluate the quality of each article, ensuring they are free from bias, valid, and reliable [12]. This appraisal tool assessed each article using a checklist that considers researchers’ biases, the relevant criteria, and other essential components necessary to deliver high-quality articles. Each article was classified based on the JBI criteria into high, moderate, and minimal risk of bias categories, with scores below 50% indicating a high risk, 50-70% indicating a moderate risk, and above 70% indicating a low risk. The researchers independently reviewed the articles and discussed their results collaboratively. Through thorough discussion and analysis, the researchers agreed that each of the five articles scored 70% or higher based on the JBI scoring, thereby qualifying all four articles for inclusion in the final scoping review.

**Figure 1:**
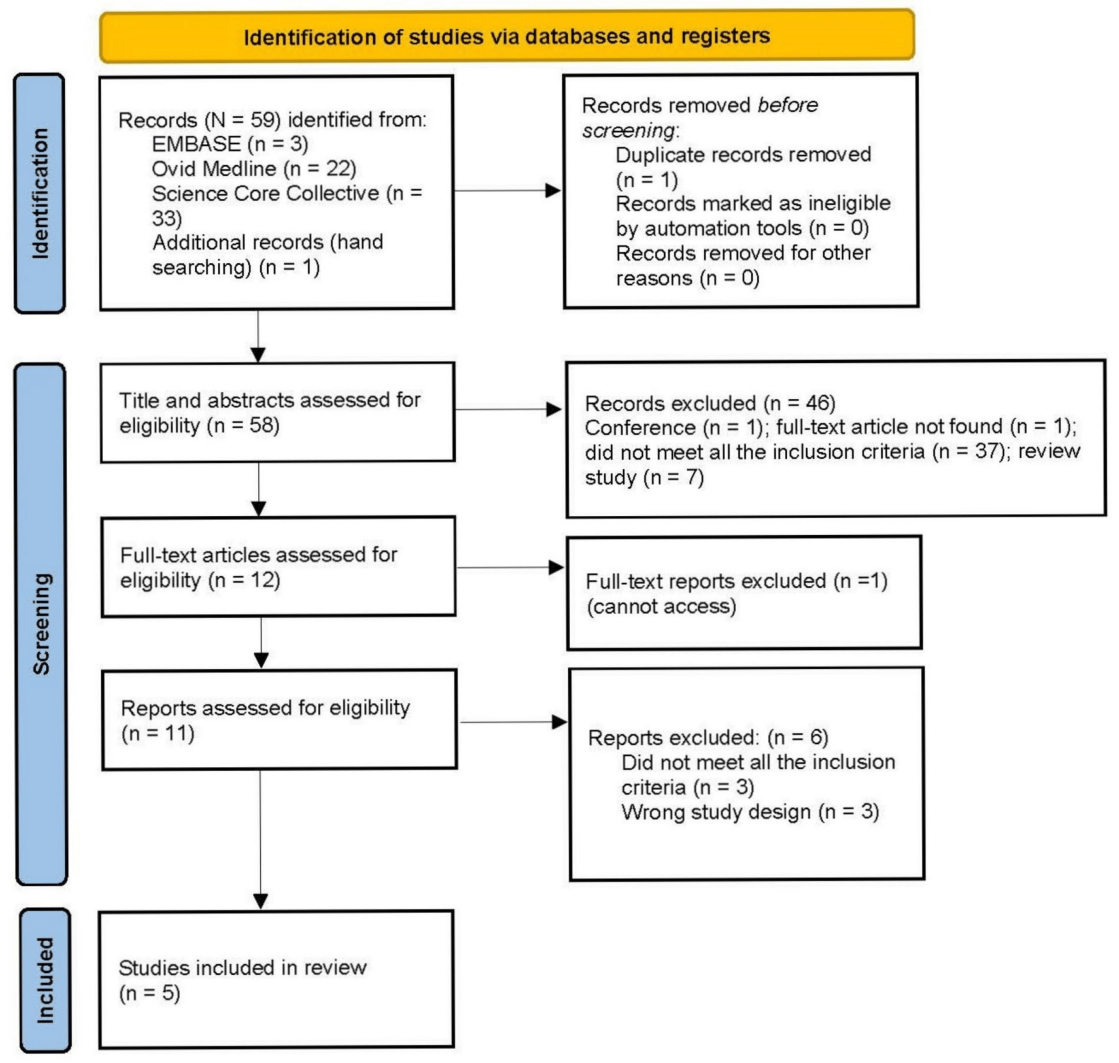
PRISMA Flow Diagram PRISMA: Preferred Reporting Items for Systematic Reviews and Meta-Analyses

The selection results were reported and are presented in Figure 1 using the Preferred Reporting Items for Systematic Reviews and Meta-analyses extension for scoping reviews (PRISMA-ScR) flow diagram [13].

Results

AI and Medication Safety

Across multiple studies, AI-based and web applications have demonstrated substantial improvements in detecting potentially inappropriate medications (PIMs) and identifying harmful drug-drug or drug-disease interactions. Studies in Turkey and China indicated that AI-driven systems could identify such interactions and inappropriate drugs with far greater speed and accuracy than manual review. A study from the Republic of Türkiye showed that the creation of a web application was able to detect inappropriate drugs, DDIs, and drug-disease interactions about 100 times faster than a manual literature review [14]. Similarly, Yılmaz et al.’s study reported that implementing a web assistant effectively eliminated inappropriate DDIs in older patients in the Republic of Türkiye with polypharmacy [15].

Moreover, the implementation of AI-assisted tools in clinical settings has resulted in a significant reduction in inappropriate drug use and the elimination of contraindicated drug combinations among older adults. One study examining AI-assisted detection of PIMs in Chinese outpatient hospitals found that the Classifier Chain and CatBoost model outperform other approaches in both speed and accuracy [16]. Another study further demonstrated the need for AI use in polypharmacy, concluding that their AI tool detected potentially inappropriate drug use in 86.9% of geriatric patients in the nursing home studied. After the implementation of the AI tool combined with physician decisions, there were no remaining contraindicated DDIs in the study [15]. These findings show the potential for these tools to be implemented in a bedside inpatient setting to prevent PIMs.

AI and Medication Adherence

Beyond medication review, AI has shown promise in promoting medication adherence. ML and audio-based activity recognition systems have been evaluated for their ability to remind and encourage older adults to take their medications as prescribed. One study found that not only are the AI tools accurate, but also useful and user-friendly, as described by all participants in the study [17]. These well-received interventions indicated technological effectiveness as well as the usability and acceptability of AI tools in supporting daily medication management.

AI in Risk Prediction and Patient Stratification

AI-assisted predictive modeling has also been used to identify patterns between multimorbidity and polypharmacy. One study found patient subgroups at particularly elevated risk for complications, such as those with overlapping cardiovascular diseases [18]. These insights may help clinicians proactively tailor interventions to those most likely to benefit, optimizing resource allocation and improving health outcomes.

Synthesis of Findings and Identified Gaps

Collectively, these studies highlight the efficiency, accuracy, and potential clinical utility of AI-based tools in various aspects of polypharmacy management, including safer prescribing, improved patient adherence, and more precise risk stratification. Despite these promising findings, an important gap in the literature is the lack of research on the long-term impact of AI-assisted interventions on clinical outcomes such as morbidity, mortality, and healthcare utilization. Future research should explore whether AI-driven medication management leads to sustained improvements in patient health and healthcare utilization over time.

Additionally, the absence of internationally standardized criteria for PIMs and methodological variations between studies limit the generalizability of findings. For example, Hu et al. used the American criteria for PIMs, which include some PIMs that are rare or absent in China and could have potentially left out PIMs that are present in China but rare or absent in America [16]. Creating internationally accepted PIM criteria would allow for more consistent results with better accuracy.

Notably, despite differences in geographic location and AI methodologies, all studies reviewed yielded significant results supporting AI’s effectiveness in detecting PIMs. Regardless of the AI program used or the geographic location, all studies had significant results that supported the use of AI in detecting PIMs. Although further research is needed before the regular use of AI in PIMs is implemented, the results in these studies present the importance of using AI in detecting PIMs and call for continued efforts in the body of related research. A summary of the articles included in this review is reported in Table 2.

**Table 1:** Final Summary Table of Included Articles RDU: rational drug usage; AI: artificial intelligence; PIM: potentially inappropriate medicines; EU European Union; US: United States; MAQ: Medication Adherence Questionnaire; SHRI: Self-Reported Habit Index; SUS: System Usability Scale; CHI: Council of Health Insurance

Author(s)	Country of Study	Study Aims	Sample and Setting	Methods	Main Findings	Limitations	Conclusions
Akyon et al. 2023 [14]	Republic of Türkiye	To create an auxiliary tool to reduce the complications of polypharmacy and support rational drug use (RDU) in geriatric patients.	1,203 active pharmaceutical agents licensed from the Türkiye Pharmaceuticals and Medical Devices Agency 2022 E-Prescription Drug List.	First phase: an up-to-date and comprehensive auxiliary tool as a reference method was generated with interaction information of 430 most used drug agents and chronic diseases in geriatrics. Then, an AI-supported web application was designed to facilitate the practical usage of the tool. Finally, data from a cross-sectional, observational, single-center study were used for the rate and time of PIMs and drug interaction detection with the web application.	PIM coverage rate with the proposed tool was 75.3%, the PIM coverage rate of EU (7)-PIM, US-FORTA, TIME-to-STOPP, Beers 2019, STOPP, Priscus criteria in the web application database, respectively (63.5%–19.5%) from the highest to the lowest. Detection time of PIMs, drug-drug, and drug-disease interactions using the web application decreased to 33.8 seconds from 2278 seconds on average. The web application is two times more comprehensive than the most comprehensive criteria for inappropriate drugs in geriatric patients and five times more comprehensive than the criteria with the lowest coverage in determining PIM.	It is not known how long patients were using the medications. Lack of sufficient and heterogeneity of criteria for PIM, drug-drug interaction, and drug-disease interactions make it hard to evaluate RDU in clinical practices.	The web application detects inappropriate drugs, drug-drug interactions, and drug-disease interactions approximately 100 times faster than a manual literature review, which can reduce time constraints problems in clinical practice. Potential socio-economic contributions of the web application in reducing multi-morbidity and mortality.
Hu et al. 2023 [16]	China	Identify PIMs through a machine learning-based warning model. The study attempted to detect PIMs in Chinese outpatient hospitals in elderly patients. By testing and comparing many models, the study determined the most effective model to detect PIMs.	7,115 tertiary outpatient hospitals in China. Older adults with a mean age of 78.69 ± 8.29 years (range 65 to 119 years)	Retrospective observational study determining PIMs through machine learning. The study used data retrospectively from outpatient hospitals in China and ran the pharmacological data through six different machine learning models and three problem transformation methods. The performance of the models was evaluated based on accuracy, precision, recall, and F-1 score metrics.	Among 11,741 older patient prescriptions, 5816 PIMs were reported from 4038 (34.39%) patient prescriptions. The Classifier Chain + CatBoost model was found to be the best-performing system for automated PIM detection. This model had the highest accuracy value (97.83%), recall value (89.34%), F1 value (90.69%), and ss Acc value (97.79%). This was the selected model to be used in China to warn of PIMs in the geriatric population.	The Beers criteria, which were used as the criteria to determine PIMs, were developed by the American Geriatrics Society. Some PIMs in these criteria are rare or absent in China. Patients’ personal information (e.g., weight, smoking history) cannot be obtained, which can lead to the detection rate of PIM prescriptions being underestimated or overestimated.	Classifier Chain + CatBoost outperforms other models in detecting PIMs. This model or future versions of it could be used to quickly and accurately identify PIMs, simplify the manual evaluation, and reduce heterogeneity between different evaluators. These machine-based learning algorithms for detecting PIMs can be implemented at the bedside to improve inpatient safety in the future.
Rodriguez et al. 2021 [17]	Mexico	To design a system based on machine learning for audio-based activity recognition using Hidden Markov Models to assist older adults in developing a consistent medication schedule by associating them with daily activities.	Two females, ages 70 and 72, respectively, with both having hypertension and other chronic illnesses. Nine females in daycare centers who have one or more chronic illnesses and take 2+ medications daily.	A 30-day observational study, followed by an in-situ assessment of the audio-based activity recognition system in the two female participants. In the nine female participants, the MAQ-8 scale and the Self Report Habit Index were administered. Then the assistance dialogues used the Wizard of Oz technique, and the Echo Dot 3rd generation implemented different scenarios for each participant according to their medications and activities they reported.	In the two female participant groups, anchor activities with audible manifestation accuracy were 79% for subject 1 and 97.6% for subject 2. In the nine female participants group, most reported a low or medium perceived adherence (MAQ-8 score = 2-6), and two participants perceived they had a strong medication habit formation (SRHI score > 4). 8/9 participants considered the most useful function of the conversational agent assistance to be reminding them to take the medications correctly. High user satisfaction level using the Voice Assistant, with SUS score being 80.1.	Small sample size, along with the big discrepancy between the two female participants’ accuracy levels (79% vs 97.6%). A lack of male participants, thus preventing the results from the study from being generalized. Limitations of one audible activity per time, as there were scenarios where subjects performed 2+ activities at the same time, or multiple individuals performed the same activity at the same time.	Using anchor activities with an audible manifestation via machine learning yields high accuracy. The usability assessment of the nine participants via the Wizard of Oz technique results in the participants viewing the system as useful and easy to useful, thus they could use it to adhere to their medication regimes better.
Stafford et al. 2021 [18]	Spain	To identify multimorbidity and polypharmacy patterns for the elderly population in Catalonia. The aim of this study was to determine combined patterns of multimorbidity and polypharmacy in the Catalan population 65–99 years of age through a machine-learning soft clustering technique that incorporates the research team’s mapping of chronic disease and drug associations.	916,619 individuals aged 65-99 in Catalonia.	Cross-sectional study of electronic health records from 2012. Then, a mapping process to link chronic diseases to drug categories, and finally, a soft clustering technique on the final mapped categories.	93.1% of individuals met the authors’ criteria for multimorbidity and 49.9% for polypharmacy. Seven clusters were identified: one non-specific (Cluster 1) and six specific, corresponding to diabetes (Cluster 2), neurological and musculoskeletal, female dominant. (Clusters 3 and 4) and cardiovascular, cerebrovascular, and renal diseases (Clusters 5 and 6), and multi-system diseases (Cluster 7). Cluster 1: no overrepresented diseases, low O/E ratios, and low levels of multimorbidity (81.74%) and polypharmacy (15.38%). Cluster 2: diabetes drug group having O/E exceeding threshold (2.15). Clusters 3 and 4: predominantly females. Cluster 5: high rates of peripheral vascular disease and ischemic heart disease. High polypharmacy rates (83.65%). Cluster 6: high rate of polypharmacy (83.54%) with exclusivity for A-Fib (5.99) and heart failure (5.7). Cluster 7: the smallest percentage of the population (5.41%), but multiple overrepresented diseases in multiple systems.	Some individuals were excluded because they sought care outside of CHI governance. Hospital drugs were not included and recorded via invoices. The fuzzy c-means clustering technique is an unsupervised and exploratory method.	The study found that clusters 2-6 provided recognizable patterns between multimorbidity and polypharmacy, with higher rates in females.
Yılmaz et al. 2023 [15]	Republic of Türkiye	To evaluate nursing home residents’ medication regimens using an AI RDU web assistant. This RDU web assistant was developed by researchers to avoid unnecessary use of medication.	99 nursing home residents in Turkey. The mean age of the participants was 79.81 ± 7.64 years. 88.9% of participants were taking more than one prescribed medication.	An analytical cross-sectional study. A web-based AI tool was used to detect PIMs. The web-based tool analyzed the patients’ medications based on six established PIM criteria. It evaluated drug-drug interactions, drug-disease interactions, and contraindications.	88.9% of patients used polypharmacy. Potentially inappropriate drug use was detected in 86.9% of geriatric patients. The most common potentially inappropriate drug use was proton pump inhibitors at a rate of 64%. There was a significant difference in the number of drugs used by patients before and after the assessment with the AI web assistant.	Some drugs only become inappropriate after a set amount of exposure time; for example, proton pump inhibitors become inappropriate after 8 weeks. This study assumed that patients were using all their listed drugs for a long time. Additionally, the sample size was not large enough to make broad generalizations.	Most patients in the nursing home had polypharmacy and potential drug-drug interactions. There was inappropriate drug use in most patients over 65. There were no contraindicated drug-drug interactions remaining following the web assistant and physician decision. Web assistant was also able to decrease drug costs by 10% due to the discontinuation of drugs due to inappropriate drug use. The web application supported by AI has many potential benefits in limiting PIM and assisting physicians, especially family physicians.

Discussion

Medication Safety: Potential and Challenges

AI is poised to serve as a transformative instrument in improving medication safety for patients with polypharmacy, particularly among older adults who are at elevated risk for adverse drug events. The studies included in this review demonstrate that AI systems possess the capacity to identify PIMs and complex drug-drug or drug-disease interactions with a degree of speed and precision that substantially exceeds manual review. By providing clinicians with timely, actionable information, these technologies can help reduce the incidence of preventable medication-related harms and support safer prescribing practices. In clinical implementation, however, the effectiveness of AI depends on seamless integration with electronic health record systems, robust validation across varied healthcare environments, and sustained surveillance to ensure performance as new pharmacotherapies emerge. Barriers such as clinician skepticism, alert fatigue, and the risk of overreliance on automated decision support must be addressed through targeted training and thoughtful governance. Furthermore, the expansion of AI-driven surveillance brings ethical considerations related to patient privacy and data security to the forefront. A strong regulatory framework, coupled with ongoing professional education, will be required to ensure that the adoption of AI technologies enhances patient safety while safeguarding trust and confidentiality.

Medication Adherence: Usability and Acceptance

The use of AI to promote medication adherence presents an important opportunity to address one of the most persistent challenges in the care of older adults with multiple chronic conditions. AI-based reminders, personalized patient education, and real-time monitoring can address common barriers to adherence, such as forgetfulness, misunderstanding, or lack of motivation. When tailored to patients’ cognitive and functional capabilities, these tools may empower individuals to participate more actively in their care and enable clinicians to intervene proactively in response to emerging adherence issues. Nonetheless, disparities in digital literacy, technology access, and patient engagement could limit the reach and effectiveness of these interventions. To maximize clinical benefit and minimize inequity, future efforts should emphasize inclusive design, integration with traditional support systems, and co-development with end users. Continuous evaluation that is focused on user satisfaction, security of health information, and sustained engagement will be important to ensure that AI-facilitated adherence interventions result in meaningful improvements in health outcomes without exacerbating disparities or contributing to technology fatigue.

Risk Prediction: From Big Data to Bedside Impact

AI-based risk prediction and patient stratification hold considerable potential to optimize care for individuals with polypharmacy by enabling clinicians to identify patients at the greatest risk for medication-related complications. Predictive analytics can facilitate more precise allocation of healthcare resources and support dynamic, individualized care planning as patient conditions evolve. However, the reliability and fairness of these models are contingent upon the representativeness and quality of the data used for algorithm development and validation. Failure to account for demographic or clinical diversity in training data may perpetuate or amplify healthcare disparities. Transparent, explainable algorithms and regular auditing for bias are critical to ensuring ethical and effective deployment. Importantly, AI-generated risk scores should complement, rather than replace, clinical expertise and must be accompanied by clear protocols for clinician response to avoid overtreatment, unnecessary alarm, or stigmatization of high-risk groups. The goal should be to enhance, not supplant, the nuanced judgment that defines effective patient-centered care.

Implications for Clinical Practice

The incorporation of AI into clinical workflows presents a paradigm shift in medication management, particularly for older populations. AI has demonstrated utility in processing medical texts, electronic health records (EHRs), and large-scale research data to extract clinically salient information. Furthermore, AI’s application can extend to diagnostic imaging, where it assists in analyzing CT scans, MRIs, and X-rays in cardiology, oncology, and radiology. In the context of medication management, AI can be integrated into EHR systems and prescribing software to preemptively identify PIMs, DDIs, and contraindications before prescription finalization. By flagging potential adverse effects, AI can provide early alerts to physicians, allowing for a more proactive approach to patient safety. Nevertheless, AI-generated recommendations necessitate physician validation to ensure alignment with individualized patient needs and clinical judgment.

Clinician oversight is an indispensable component of AI deployment, and AI should function as a support mechanism rather than supplant human decision-making. Similarly, a clinician-centered AI tool for diagnostic radiology may underscore the importance of physician involvement. Ethical considerations surrounding AI autonomy further highlight the necessity of human oversight. Beyond decision support, AI holds promise in improving adherence through automated reminders, patient education, and continuous monitoring, thereby mitigating adverse drug events and enhancing therapeutic outcomes in older adults.

Limitations of the Individual Sources of Evidence

The individual studies included in this review possess several noteworthy limitations that impact the strength and applicability of their findings. A common issue was the use of small sample sizes and demographically homogenous study populations, limiting the generalizability of results to broader or more diverse groups. Some studies lacked adequate representation of different genders and underrepresented groups, further constraining external validity. Additionally, there was considerable heterogeneity in the criteria used to define PIMs, DDIs, and drug-disease interactions, making cross-study comparisons challenging and contributing to inconsistencies in reported outcomes. In certain cases, studies relied on criteria such as the Beers list, which may not fully reflect medication risks in non-American populations, leading to potential misclassification of PIMs. Other methodological concerns included the inability to accurately track the duration of medication use, the exclusion of patient-level clinical information such as weight or comorbidities, and the omission of medications obtained outside of institutional oversight. Studies utilizing ML for medication adherence also reported limitations in activity recognition, such as difficulty distinguishing overlapping activities or scenarios involving multiple individuals. The reliance on unsupervised clustering techniques in some predictive modeling studies introduces additional uncertainty regarding the reproducibility and clinical relevance of their findings. Taken together, these limitations underscore the need for larger, more diverse, and methodologically rigorous studies to clarify the impact of AI interventions in polypharmacy management.

Methodological constraints, including small demographically homogenous sample sizes in certain studies, further limit extrapolation to broader populations. Notable gaps of pertinent information include an inability to track medication duration in one study and the exclusion of patients’ personal information and out-of-hospital data.

Limitations of the Review

While this review provides valuable insights into the integration of AI in polypharmacy management for older adults, several limitations must be considered. One limitation of this scoping review is the relatively small number of eligible studies included, which may restrict the breadth of insights and limit the generalizability of findings across different healthcare settings or populations. Due to the novelty of AI, current research predominantly focuses on short-term effects of reducing PIMs and the cost of unnecessary medications, while long-term assessments regarding patient quality of life, morbidity, and mortality following AI-assisted intervention are absent. Furthermore, the lack of consistent international criteria for defining PIMs and the variations in prescription guidelines utilized in each country pose challenges to AI’s generalizability and application [14, 16].

Future Directions for Research

Further exploration of AI-driven personalized pharmacotherapy could improve medicine approaches, reducing adverse effects while optimizing therapeutic efficacy. Accounting for individual comorbidities, pharmacokinetics, and pharmacogenomic profiles will optimize resource utilization and management strategies targeted toward each patient’s specific needs. The integration of AI in managing multiple pharmacological therapies shows significant potential, but additional research is needed to ensure seamless integration into the clinical workflow. Longitudinal studies evaluating AI’s sustained impact on patient morbidity, mortality, and quality of life are imperative to investigate before the application of these tools.

Despite the significance of managing opioid use for chronic pain among older adults, limited research explores AI’s potential role in mitigating opioid-related risks, including overdose, drug-drug and drug-disease interactions, and dependency. Addressing this research gap would provide critical insights into AI’s applicability in opioid prescribing and deprescribing strategies.

Standardization of AI-assisted medication review tools is critical for consistency, reliability, and widespread integration into healthcare settings. Establishing uniform guidelines and defining fundamental concepts such as PIMs demands focused research to provide dependable and comprehensive healthcare recommendations across a variety of healthcare settings. Investigation of AI’s effectiveness across these diverse settings, including nursing homes, primary care facilities, and hospitals, must also be explored, as each presents unique situations and patient populations.

## Conclusions

AI has the potential to influence a progressive change in polypharmacy management, offering a scalable solution for enhancing medication safety in older adults. AI-driven tools have demonstrated some efficacy in identifying PIMs, optimizing prescribing practices, and reducing overall clinician workload. However, it is suggested that AI should be conceptualized as a complementary asset rather than a substitute for physician expertise. While AI can process complex medication regimens with unparalleled efficiency, final clinical decisions must remain within the purview of trained medical professionals who can integrate contextual patient factors, clinical nuance, and ethical considerations into the decision-making process. Further research and rigorous validation may be warranted before AI-driven medication management becomes standard practice. Future investigations should focus on AI’s long-term impact on patient outcomes, its role in personalized pharmacotherapy, and ongoing challenges related to algorithmic transparency, bias mitigation, and regulatory oversight. As AI continues to evolve, its integration into healthcare must be guided by methodical evaluation, stringent oversight, and interdisciplinary collaboration to ensure its safe and effective deployment in patient care.

## Appendices

**Table 2:** Article Search Strategy

Database Search Translations		
EMBASE – 9/15/2024		
1	polypharmacy	31975
2	(polypharmacy)/br AND (('artificial intelligence')/br)	118
3	(polypharmacy)/br AND (('artificial intelligence')/br) AND (('older adults')/br)	11
4	(polypharmacy)/br AND (('artificial intelligence')/br) AND ([Controlled Clinical Trial]/lim OR [Randomized Controlled Trial]/lim)	3
Ovid MEDLINE(R) ALL – 9/15/2024		
1	polytherap* OR polypharmacy OR multipharmaceutical OR multiple medication OR multiple medicine	16778
2	(polytherap* OR polypharmacy OR multipharmaceutical OR multiple medication OR multiple medicine) AND ("older adults" OR ELD*)	5506
3	"artificial intelligence" OR "machine learning" OR "machine intelligence" OR "large language model*" AND healthcare OR "medical management"	33780
4	(polytherap* OR polypharmacy OR multipharmaceutical OR multiple medication OR multiple medicine) AND ("older adults" OR ELD*) AND ("artificial intelligence" OR "machine learning" OR "machine intelligence" OR "large language model*")	22
Web of Science Core Collection – 9/15/2024		
1	polytherap* OR polypharmacy OR multipharmaceutical OR multiple medication OR multiple medicine	1024462
2	(ALL= (polytherap* OR polypharmacy OR multipharmaceutical OR multiple medication OR multiple medicine)) AND ALL= ("Older adults" AND ELD*)	4,118
3	((ALL= (polytherap* OR polypharmacy OR multipharmaceutical OR multiple medication OR multiple medicine)) AND ALL= ("Older adults" AND ELD*)) AND ALL=("artificial intelligence" OR "machine learning" OR "machine intelligence" OR "large language model*")	33
